# Are Invasive Procedures and a Longer Hospital Stay Increasing the Risk of Healthcare-Associated Infections among the Admitted Patients at Hiwot Fana Specialized University Hospital, Eastern Ethiopia?

**DOI:** 10.1155/2020/6875463

**Published:** 2020-03-31

**Authors:** Moti Tolera, Dadi Marami, Degu Abate, Merga Dheresa

**Affiliations:** ^1^School of Public Health, College of Health and Medical Sciences, Haramaya University, PO Box 235, Harar, Ethiopia; ^2^Department of Medical Laboratory Sciences, College of Health and Medical Sciences, Haramaya University, PO Box 235, Harar, Ethiopia; ^3^School of Nursing and Midwifery, College of Health and Medical Sciences, Haramaya University, PO Box 235, Harar, Ethiopia

## Abstract

**Background:**

Healthcare-associated infection is a major public health problem, in terms of mortality, morbidity, and costs. Majorities of the cause of these infections were preventable. Understanding the potential risk factors is important to reduce the impact of these avoidable infections. The study was aimed to identify factors associated with healthcare-associated infections among patients admitted at Hiwot Fana Specialized University Hospital, Harar, Eastern Ethiopia.

**Methods:**

A cross-sectional study was carried out among 433 patients over a period of five months at Hiwot Fana Specialized University Hospital. Sociodemographic and clinical data were obtained from a patient admitted for 48 hours and above in the four wards (surgical, medical, obstetrics/gynecology, and pediatrics) using a structured questionnaire. A multivariate logistic regression model was applied to identify predictors of healthcare-associated infections. A *p* value <0.05 was considered statistically significant.

**Results:**

Fifty-four (13.7%) patients had a history of a previous admission. The median length of hospital stay was 6.1 days. Forty-six (11.7%) participants reported comorbid conditions. Ninety-six (24.4%) participants underwent surgical procedures. The overall prevalence of healthcare-associated infection was 29 (7.4%, 95% CI: 5.2–10.6). Cigarette smoking (AOR: 5.18, 95% CI: 2.15–20.47), staying in the hospital for more than 4 days (AOR: 4.29, 95% CI: 2.31–6.15), and undergoing invasive procedures (AOR: 3.58, 95% CI: 1.11–7.52) increase the odds of acquiring healthcare-associated infections.

**Conclusion:**

The cumulative prevalence of healthcare-associated infections in this study was comparable with similar studies conducted in developing countries. Cigarette smoking, staying in the hospital for more than 4 days, and undergoing invasive procedures increase the odds of healthcare-associated infections. These factors should be considered in the infection prevention and control program of the hospital.

## 1. Introduction

The term “healthcare-associated infection (HCAI)” has replaced the “nosocomial” or “hospital-acquired” infection, for its occurrences as a result of medical devices such as catheters and ventilators; complications following surgical procedures, and transmission between patients and healthcare workers [1, 2]. Healthcare-associated infection is defined as a localized or systemic infection occurring in a patient during the process of care in a hospital that was not present or incubating at the time of admission. Most HCAIs become evident at 48 hours or more following admission [3, 4]. Infection may also prevail after discharge because the patient has become colonized or infected while in hospital, but the pathogen incubation period exceeds the patient's hospital stay [2].

Despite the significant improvement in treatment and advances in medical care, HCAIs are increasingly becoming a major public health problem and posing a great threat to patient safety and wellbeing in both developed and developing countries [5, 6]. Globally, more than 2–4 million people are suffering from HCAIs [7]. The burden of infection is more pronounced in developing countries due to limited financial resources and facilities that may help to reduce the transmission of pathogenic organisms [8, 9].

Extremes of age (the younger and older age), underlying diseases [10–12], longer stays in hospital, invasive medical procedures, poor aseptic techniques of healthcare workers, overcrowd, and repeated hospitalization increase the likelihood of acquiring HCAIs [13, 14]. The risk has been estimated to be 2–20 times higher in developing countries than that of developed countries with the rate of infection exceeding 25% [13]. Understanding factors associated with infection in the hospital are strategically important for devising a prevention mechanism to enhance the safety of hospitalized patients [15, 16].

In many developing countries, data about the prevalence and associated factors of HCAIs at referral hospitals are scarce. The existing literatures, particularly in sub-Saharan Africa countries, were restricted to certain wards of the hospitals [17–19]. Likewise, studies conducted in Ethiopia are mainly limited to particular sites of infections [20–22], but minimizing HCAIs within the hospitals is a key aspect of patient safety initiatives. Thus, the true picture of the problem is not well elucidated in Eastern Ethiopia. This study was aimed to identify factors associated with clinically confirmed HCAIs among patients admitted at Hiwot Fana Specialized University Hospital, Eastern Ethiopia.

## 2. Materials and Methods

### 2.1. Study Setting and Design

A cross-sectional study was conducted in Hiwot Fana Specialized University Hospital from March 2017 to July 2017. The hospital is found in Harar town which is located 525 km from Addis Ababa, Ethiopia. Hiwot Fana Specialized University Hospital is the grand teaching hospital of Haramaya University. The hospital accepts referral patients from different parts of the eastern Ethiopian regions and provides healthcare services with annual average admission of over 20,000 patients. The hospital provides surgical, medical, obstetrics, gynecology, nutrition, pediatric, mental health services, maternity, and outpatient/day surgery services [23].

### 2.2. Study Population and Sample Size

A total of 433 patients were investigated as described in our previously published article [24]. A patient who was admitted to the medical, surgical, obstetrics, gynecology, and pediatric wards and develops signs and symptoms of HCAIs within 48 hours or more after admission and was not incubating the same diseases on admission was included in the study. These patients who were hospitalized at the emergency ward, recovery ward, psychiatric units, maternity wards, day surgery units, and/or readmission with the previous conditions were excluded from the study. No patient was enrolled more than once.

### 2.3. Data Collection

A structured questionnaire and checklist adapted from different literatures [2, 10] were used to collect the data. Initially, the instruments were developed in English and later translated into local languages (*Amharic* and *Afan Oromo*) for the convenience of data collection. The pretest of the questionnaire was done on 5% of patients satisfying the inclusion criteria at the Dilchora Referral Hospital, Dire Dawa, Eastern Ethiopia, to check its consistency and simplicity.

The clinical diagnoses of the patients and interview were made by trained general practitioners, health officers, surgeons, pediatricians, internists, and gynecologists recruited from other health institutions. Patients were screened for signs and symptoms of HCAIs. Patients with a new occurrence of fever (>38°C) at least 48 hours after admission or a repeated episode of fever after a nonfebrile interval of 72 hours were qualified for further evaluation. The further evaluation consisted of personal data, clinical examination, the reason for hospitalization, operation status, current signs and symptoms, and invasive medical procedure. An invasive procedure is one where purposeful access to the body is gained via an incision and percutaneous puncture, where instrumentation is used in addition to the puncture needle or instrumentation via a natural orifice (include indwelling vascular lines, drug injection, blood sample collection, blood transfusions, urethral catheterization, paracentesis, endoscopes, stitching, thoracentesis, and so on). Medical records, temperature charts, surgeon notes, and laboratory reports of each participant were revised to obtain the results of complementary examinations and likely to confirm (rule in/out the HCAI) in case there is ambiguity. Patients were followed up daily for symptoms of HCAIs until they were discharged. The Centers for Disease Control and Prevention (CDC) criteria were applied to classify and define HCAIs. HCAI is defined as a localized or systemic infection that occurred at least 48 hours after admission of the patient to the hospital, for which there is no evidence for the infection was present or incubating at admission [2]. The comorbid conditions were defined when a patient presents in an index or previous admission records. Otherwise, the absence of the condition was assigned to the patient. Data collection was closely supervised by the investigators.

### 2.4. Measurement

The outcome variable was the presence of HCAIs among admitted patients. The presence of HCAIs was labeled as “yes” while the absence as “no” with a code of 1 and 0, respectively. Independent variables include sex, the patient's age, admission ward, a history of cigarette smoking, history of the previous admission, length of hospital stay, presence of comorbid conditions, invasive procedures, availability of hand washing material, and the presence of medical waste in the wards among the others. The age of the patients was grouped as <15, 15–34, 35–55, and >55 with a code of 0, 1, and 2, respectively, for descriptive analysis and <18 and ≥18 with a code of 0 and 1, respectively, for logistic regression analysis. Cigarette smoking, history of the previous admission, the presence of comorbid diseases, the presence of antiseptic for hand rubbing, invasive medical procedure, and mechanical ventilation were recorded as “yes” or “no” with a code of 1 and 0, respectively.

### 2.5. Data Analysis

To minimize errors related to the data entry, data were coded, checked, and double entered into Epidata version 2.0 (Odense, Denmark), cleaned, and exported to the Statistical Package for Social Sciences version 25.0 (SPSS, IBM Corp., Armonk, NY, USA) for analysis. Only the first HCAI occurring in a patient was considered to calculate the prevalence of HCAIs if the patient had two or more infections. Categorical variables were expressed in numbers and percentages. Bivariate and multivariate logistic regression models were used to identify the predictors of HCAI. The variables with a *p* value ≤0.25 in the bivariate analysis were further analyzed in a multivariate logistic regression model. The model of fitness was checked by Hosmer and Lemeshow, which provided evidence of fitness with a predictor test level of *p*=0.82. The finding was presented using the adjusted odds ratio (AOR) at a 95% confidence interval (CI). A *p* value <0.05 was considered statistically significant.

### 2.6. Ethical Consideration

Ethical clearance was obtained from the Institutional Health Research Ethics Review Committee of the College of Health and Medical Sciences, Haramaya University. Informed, voluntary, written, and signed consent was obtained from each participant before the commencement of the study. For those whose age was <18 years, consent was obtained from their parents/legal guardians whereas assent from the child. The information provided during the study was kept confidential. The findings of the study were not reflected in anything about individuals or persons.

## 3. Results

A total of 433 individuals took part in the study, and 39 of them were excluded from the study (23 of which refused to participate, 8 changede the ward, 5 discharged before completing data collection, and 3 died). The response rate was 394 (91%). Of the total participants, more than half, 223 (56.6%) were females. The mean (±standard deviation) age of the patients was 22.5 ± 18.3 years, ranging from 1 to 80 years. Medical, obstetrics, and/or gynecology wards constituted 205 (52%) of the participants. Of the total, 53 (6.8%) had a history of smoking. Fifty-four (13.7%) patients were reported having a history of the previous hospitalization elsewhere for either the same as the current of admission or other ailments. The median length of hospital stay after the onset of HCAIs was 6.1 days (3.2 days with no HCAIs and 5.9 days with HCAIs). Forty-six (11.7%) patients had comorbid diseases. The majority of comorbid conditions was all forms of tuberculosis (10 (21.7%)) followed by hemorrhoids (8 (7.4%)) (Table 1).

A large number of participants (96 (24.4%)) underwent surgical procedures. The majority of surgical procedures performed were cesarean section (22 (22.9%)) followed by debridement of wounds (18 (18.8%)). Among the major surgeries, 60 (62.5%) had clean-contaminated surgery (Table 2).

Out of the total 394 patients enrolled, 29 (7.4%, 95% CI: 5.2–10.6) had HCAIs. Three patients died because of HCAIs, making a case fatality rate of 10.3%. Admission in the surgical ward, cigarette smoking, history of the previous admission, stay for more than 4 days in the hospital after the onset of HCAIs, comorbid condition, and underwent invasive procedures were associated with HCAIs in bivariate logistic regression analysis at a *p* value ≤0.25. In multivariate analysis, patients who smoke cigarettes (AOR: 5.18, 95% CI: 2.15–20.47) had higher odds of HCAIs compared to their counterparts. The odds of developing HCAI among patients who stay in the hospital for more than 4 days after the onset of HCAIs were 4.3 times higher than their counterparts (AOR: 4.29, 95% CI: 2.31–6.15). The odds of developing HCAI among patients who had invasive procedures since admission was 3.6 times higher compared to patients without invasive procedures (AOR: 3.58, 95% CI: 1.11–7.52) (Table 3).

## 4. Discussion

The overall prevalence of clinically confirmed health-associated infection (7.4%) in this study was in agreement with the finding reported in Iran (9.4%) [25], but much higher than that reported from Lambarene, Gabon (1.6%) [19]. However, it was lower compared to the findings reported from other developing countries such as in Jimma, Ethiopia (19.1%) [15]; Gondar, Ethiopia (14.9%) [22]; and Lahore, Pakistan (11.3%) [26]. The observed difference can be explained by the variation in the studied ward as this study could not include patients from an emergency and recovery wards and maternity and day surgery units which may contribute to increasing the prevalence of HCAIs. The high patient load, level of awareness about infection prevention, lack of aseptic practices by janitors and care providers, nonadherence to safe practices by health workers, shortage of water supply, ineffective diagnostic policies, and poor laboratory backup [27] and sample size could also contribute to the observed differences.

The prevalence of HCAIs in the surgical wards in this study (37.9%) was higher compared with the findings from Jimma, Ethiopia (29.6%) [15]; Riyadh, Saudi Arabia (13.3%) [28]; and Gondar, Ethiopia (10.2%) [22]. Infection prevention, particularly in surgical wards, could not be ignored since the infection is critical and easily spreads throughout the hospital making the surgical wards a focal point [29]. The history of surgical intervention, the nature of surgical interventions, longer duration of surgical procedures, and the overcrowding of bed in wards may contribute to the variation [18, 20]. The observed discrepancy may also be due to the differences in sample size, methods adopted, and inclusion criteria.

Cigarette smoking is one of the avoidable causes of local and systemic adverse effects on the immune system, respiratory tract, skin, and soft tissues [30]. Furthermore, cigarette smoking has an immune suppressive effect on the patients and promotes microbial virulence and antibiotic resistance, which together predisposes the patients to various HCAIs [30, 31]. According to the World Health Organization (WHO), smoking causes about 9% of all deaths worldwide [31]. In this study, the odds of developing HCAIs were 5-fold higher in cigarette smokers compared to nonsmokers. A number of studies reported that cigarette smoking increases the likelihood of having an infection such as invasive pneumococcal disease, periodontitis, meningococcal disease, tuberculosis, surgical wound infections, and postoperative mediastinitis [30–32] although they failed to give statistical significance supporting these findings.

The onset of HCAI extends the length of hospital stay [33, 34] and also affects the prevalence of HCAI due to cross-contamination and the patient's susceptibility to infection [4, 29]. In this study, the median length of hospital stay after the onset of HCAIs was 6.1 days and patients who were admitted for more than 4 days in the hospital after the onset of HCAI had 4-fold odds of HCAIs. This may also support the argument that prolonged hospital admission increases the likelihood of HCAI. The finding was in agreement with most studies [1, 35]. This may partly be explained by the hypothesis that patients developing HCAIs have more comorbidities which lead to a longer inpatient stay [1, 36, 37].

In the healthcare system, life-saving invasive procedures using equipment are found to increasingly threaten patients [25, 38]. This is related to the comorbidity between patients carrying the device and the greater possibility of entry of pathogenic organisms in these patients, thereby causing the infection [39]. In this study, the odds of infection were greater by 3.6 times among patients undergoing invasive procedures than their counterparts. A study conducted in Poland showed a positive correlation between exposure to invasive procedures and HCAIs [38]. The finding was also supported by other studies conducted elsewhere [38, 39].

Even though there was no association between the absence of hand washing basin, absence of antiseptic for hand rubbing, absence of medical waste container and poor mechanical ventilation, and HCAIs in the current study, there has been a number of reports that describe the risk of these factors [3, 15]. Hand washing habit using soap [12], use of protective clothing, good personnel and hospital hygiene, adequate management of soiled linen, proper management of waste in the hospital, aseptic techniques in the operating theatre, and isolation of highly contagious patients are cited as important precaution to prevention and control of HCAI in the hospital [3, 27].

The strength of this study is that it utilized data originating from real patients. The revision of detailed clinical data is used to support the selection of participants, in addition, to prospectively follow the cases until discharge by implementing the United States National Health and Nutrition Examination Survey and the CDC criteria. To increase the genuineness of the data, questions were formulated in such a way to remember the event with indexed questions and training provided before the study in order to standardize the procedures for data collection. Therefore, evidence from this study can be treated as a benchmark for similar resource-limited settings, provides clues for the development of future interventions, helps practitioners, prioritizes interventions, and targets future prevalence surveillance to reduce the risk of infection in the hospital. However, the true burden of HCAIs could not be captured as this study is a cross-sectional study and leaves out patients who may potentially develop HCAIs after discharge. The lack of assessing risk factors related to the infection prevention practices of health professionals, equipment sterilization methods, body mass index, prophylaxis provided prior to surgery, and recall bias could also be the limitation of this study.

## 5. Conclusions

The results of this study revealed a substantial threat of HCAIs in hospitalized patients. Although the overall prevalence of HCAIs was comparable with other reports from developing countries, there are several areas that need to be improved. The hospital needs to make a concrete effort to minimize HCAIs by following strict aseptic invasive procedures, effective methods of sterilization, working towards the reduction of hospital stay, and availing hand washing basins and antiseptics in the wards. Cigarette smokers should be counseled by attending health professionals on the health effect of smoking. A prospective longitudinal study is recommended in order to describe HCAIs and risk factors more broadly.

## Figures and Tables

**Table 1:** Characteristics of the study patients admitted to the Hiwot Fana Specialized University Hospital, Harar, Eastern Ethiopia, 2017.

Characteristics of participants	*n* (%)
Sex	
Male	171 (43.4)
Female	223 (56.6)
Age (in years)	
<15	129 (32.7)
15–34	156 (39.6)
35–55	81 (20.6)
>55	28 (7.1)
Admission ward	
Medical	102 (25.9)
Obs/Gyn	103 (26.1)
Pediatric	90 (22.9)
Surgical	99 (25.1)
History of smoking	
Yes	53 (6.8)
No	219 (28.2)
Previous history of admission	
Yes	54 (13.7)
No	340 (86.3)
Length of hospital stay (in days)	
≤4	155 (39.3)
>4	239 (60.7)
Comorbid conditions	
Yes	46 (11.7)
No	348 (88.3)
Types of comorbidity (*n* = 46)	
Diabetes	7 (15.2)
Hemorrhoids	8 (17.4)
Hypertension	7 (15.2)
Boils	3 (6.5)
Cancer	7 (15.2)
Tuberculosis	10 (21.7)
HIV positive	4 (8.7)

Obs/Gyn: obstetrics/gynecology.

**Table 2:** Surgical profile of the study patients admitted to the Hiwot Fana Specialized University Hospital, Harar, Eastern Ethiopia, 2017.

Surgery conditions	*n* (%)
Surgery	
Yes	96 (24.4)
No	297 (75.6)
Types of surgical procedure (*n* = 96)	
Cesarean section	22 (22.9)
Debridement of wound	18 (18.8)
Laparotomy	9 (16.7)
Eye surgery	7 (9.4)
Appendectomy	6 (6.3)
Myomectomy	4 (4.2)
Hysterectomy	3 (3.13)
Lipoma incision	3 (3.13)
Amputation	3 (3.13)
Others^*∗*^	21 (21.9)
Level of surgical site contamination (*n* = 96)	
Clean	24 (25)
Clean-contaminated	60 (62.5)
Contaminated	12 (12.5)

^*∗*^Others: anterior colporrhaphy, cholecystectomy closure, hydrocelectomy, splenectomy, vulvectomy, hemorrhoidectomy, tonsillectomy, breast biopsy, hysteroscopy, and free skin graft.

**Table 3:** Factors associated with HCAIs among the study participates admitted in Hiwot Fana Specialized University Hospital, Harar, Eastern Ethiopia, 2017.

Characteristics of patients	HCAIs		Crude OR (95% CI)	Adjusted OR (95% CI)
	Yes, *n* (%)	No, *n*(%)		
Sex				
Male	14 (48.3)	157 (43.0)	1	
Female	15 (51.7)	208 (57.0)	1.24 (0.58–2.64)	
Age (in years)				
<18	10 (34.5)	112 (30.7)	1	
>18	19 (65.5)	253 (69.3)	1.40 (0.64–3.06)	
Admission ward				
Medical	5 (17.2)	97 (26.6)	1	1
Obs/Gyn	6 (20.7)	97 (26.6)	0.83 (0.25–2.82)	3.49 (0.80–15.19)
Pediatric	7 (24.1)	83 (22.7)	0.54 (0.17–1.69)	2.04 (0.46–9.01)
Surgical	11 (37.9)	88 (24.1)	0.45 (0.15–1.38)^*∗*^	1.30 (0.18–9.31)
History of smoking cigarettes (*n* = 272)				
No	7 (24.1)	212 (83.5)	1	1
Yes	11 (61.1)	42 (16.5)	7.93 (2.91–21.64)^*∗*^	5.18 (2.15–20.47)^*∗*^^*∗*^
History of previous admission				
No	21 (72.4)	319 (87.4)	1	1
Yes	8 (27.6)	46 (12.6)	2.64 (1.11–6.31)^*∗*^	5.04 (0.72–35.17)
Length of hospital stay (in days)				
<4	20 (69.0)	135 (37.0)	1	1
>4	9 (31.0)	230 (93.2)	3.79 (1.68–8.55)^*∗*^	4.29 (2.31–6.15)^*∗*^^*∗*^
Comorbid conditions				
No	22 (75.9)	326 (89.3)	1	1
Yes	7 (24.1)	39 (10.7)	2.66 (1.07–6.63)^*∗*^	3.33 (0.13–32.93)
Presence of antiseptic for hand rubbing				
Yes	10 (34.5)	95 (26.0)	1	
No	19 (65.5)	270 (74.0)	1.50 (0.67–3.33)	
Invasive medical procedures				
No	12 (41.4)	253 (69.3)	1	1
Yes	17 (58.6)	112 (80.2)	2.42 (1.13–5.18)^*∗*^	3.58 (1.11–7.52)^*∗*^^*∗*^
Mechanical ventilation				
Yes	8 (27.6)	108 (29.6)	1	
No	21 (72.4)	257 (70.4)	0.91 (0.39–2.11)	
Presence of hand washing material in wards				
Yes	9 (31.0)	107 (29.3)	1	
No	20 (69.0)	258 (70.7)	1.09 (0.48–2.46)	
Presence of medical waste containers in wards				
Yes	19 (65.5)	223 (61.1)	1	
No	10 (34.5)	142 (38.9)	1.21 (0.55–2.68)	

OR: odds ratio; CI: confidence interval; Obs/Gyn: obstetrics/gynecology; *p* value ≤ 0.25^*∗*^; *p* value < 0.05^*∗∗*^.

## Data Availability

The SPSS data used to support the findings of this study are included within the article.

## Abbreviations

AOR: Adjusted odds ratio
CDC: Centers for Disease Control and Prevention
CI: Confidence interval
HCAIs: Healthcare-associated infections
WHO: World Health Organization.

## Supplementary Materials

Supplementary Materials Supplementary Materials. Appendix 1.4: data collection tools. Appendix 1.5: checklist for assessment of environmental risk factors and IPPS.
