# Supplementary material for: Are Invasive Procedures and a Longer Hospital Stay Increasing the Risk of Healthcare-Associated Infections among the Admitted Patients at Hiwot Fana Specialized University Hospital, Eastern Ethiopia?
Source: Adv Prev Med. 2020 Mar 31;2020:6875463. doi: 10.1155/2020/6875463 (PMC7150733; doi:10.1155/2020/6875463)
Supplement: Supplementary Materials — Appendix 1.4: data collection tools. Appendix 1.5: checklist for assessment of environmental risk factors and IPPS. [file 6875463.f1.docx]

## Appendix 1.4. Data collection tools

Serial number……………………… ward specialty ……………………..

Date of interview ………………………. Registration number ………………

**History sheet**

| Part I Socio economic profile (ማህበራዊና እኮኖሚያዊ ሁኔታ) haala qabeenyaa fi hawaasummaa | | | | | | | | | | |
| --- | --- | --- | --- | --- | --- | --- | --- | --- | --- | --- |
| Code | | **Questions (ጥያቄዎች) gaaffiilee** | **Response (መልስ) Deebii** | | | | | | **Skip(እለፍ) Darbi** | |
| **101** | | Age (ዕድሜ) Umurii | ________________ | | | | | |  | |
| **102** | | Sex (ጾታ) Saala | 1. Male (ወንድ) Dhiira 2. Female (ሴት) Dhalaa | | | | | |  | |
| **103** | | Education (የትምህርት ደረጃ) Sadarkaa barnootaa | 1. Un educated (ያልተማረ) kan baranne 2. Informal education (መደበኛ ያልሆኔ ት/ት) Barnoota Al-idilee 3. Primary (1ኛ ደረጃ) Sadarkaa 1^ffaa^ 4. Secondary (2ኛ ደረጃ) Sadarkaa 2^ffaa^ 5. Certificate (ሰርትፊኬት) Sertifikeetii 6. Diploma and above (ዲፒሎማና ከዚያ በላይ) Dipiloomaa fi sanaa ol | | | | | |  | |
| **104** | | Occupation(የሥራ ሁኔታ) Hojii | 1. Un employed (ሥራ የለለው) Hoji-dhabeessa 2. Student (ተማሪ) Barataa 3. Daily laborer (የቀን ሰራተኛ) Hojjetaa guyyaa 4. Farmer (አርሶ አደር) Qoteebulaa 5. House wife (የቤት እመቤት) Haadha warraa 6. Private sector (የግል) Kan dhuunfaa 7. Government (የመንግሥት) Mootummaa 8. Nongovernmental organization | | | | | |  | |
| **105** | | Monthly income (ወርሃዊ ገቢ) Galii kan baatii | _______________Birr | | | | | |  | |
| Part II Assessment of Risk factors | | | | | | | | | | |
| **201** | Did you have previous history of Smoking? (ታጨስ ነበር) Tamboo xuuxxaa ? | | | | 1. Yes (አዎ) Eeyyee 2. No (አየደለም) Lakki | | | | |  |
| **202** | Did you have previous history of admission? (ከዚህ በፊት ተኝተህ ታክመሃል) kanaan dura Ciistee yaalamtee? | | | | 1. Yes (አዎ) Eeyyee 2. No (አየደለም) Lakki | | | | | if ‘No’  Skip to 207 |
| **203** | If yes how many times? (ተኝተህ ከታከምክ ስንት ጊዜ ነዉ) Yeroo meeqa ciistee yaalamte? | | | | 1. Once (አንዴ) 2. Twice (ሁለቴ) 3. >=3 | | | | |  |
| **204** | When was Last admission? (መቼ ነበር ለመጨረሻ ጊዜ ተኝተህ የታከምከዉ) Yoom ture Yeroo dhumaaf ciistee kan yaalamte? | | | | 1. Within 6 months (ከ 6ወር ወዲህ) Baatii 6 asi. 2. Within a year (አመት) Waggaa 3. > 1 year (ከዐመት በላይ) Waggaa tokkoo ol. | | | | |  |
| **205** | What is the causes of last admission? (ለመጨረሻ ጊዜ ተኝተህ የታከምከው ለ የትኛው በሽታ ነበር) Yeroo dhumaa dhukkuba kamiif ciistee yaalamte? | | | | ____________________________ | | | | |  |
| **206** | For How long did you stay in hospital? (ምን ያህል ጊዜ ሆስፒታል ተኝተህ ታከምክ) yeroo meeqaaf hopitaala ciistee yaalamte? | | | | 1. <= 3 days (ቀናት) Guyyaa 2. 4-7 days (ቀናት) Guyyaa 3. > 7 days (ቀናት) Guyyaa | | | | |  |
| **207** | Did you have one or more of the following history?  (ቀጥሎ ከተዘረዘሩት ብያንስ አንዱን አለብህ)  Kanneen armaan gaditti ibsaman keessaa yoo xiqqaate tokko ni qabdaa? | | | | 1. Diabetis Mellitus (ስኳር) Sukkaara 2. Prolonged Steroid use (ሰትሮየድ) Isterooyidii 3. Hypertension (የደም ግፊት) Danfa Dhiigaa 4. Boils (ብጉንጅ) Dhullaa 5. Cancer (ካንሰር) Kaansarii 6. Tuberculosis (የሳማባ ነቀርሳ) Daranyoo sombaa 7. Reteroviral infection (ኤች.አይ.ቪ) 8. Others specify (ሌላ ይገለጽ ) Kan biro ____________________________ | | | | |  |
| **208** | Did you have history of previous antibiotics use within one month? (ከዚህ በፊት ፀረ-ባክቴሪያ መድሃኒት ወስደህ ታዉቃለህ/ሽ)?  Dawaa farra baakteriyaa fudhattee beektaa? | | | | 1. Yes (አዎ) Eeyyee 2. No (አየደለም) Lakki | | | | | Go to 301 if ‘no’ |
| **209** | If yes, what type of antibiotics  (ወስደህ/ሽ ከነበር የትኛውን መድሃኒት)?  Yoo fudhatte Dawaa gosa kami? | | | | 1. Gentamycin (ጀንታማይሲን) 2. Ceftriaxone (ሰፍትራክሶን) 3. Ciprofloxacin (ስፕሮፍሎክሳሲሊን) 4. Metronidazole (ሜትሮንዳዞል) 5. Others (ሌላ) kan biro___________ | | | | |  |
|  |  | | | |  | | | | |  |
| **Observational check list and vital sign measurements**  Serial number……………………… ward specialty ……………………..  Date of interview ……………………….Registration number ……………… | | | | | | | | | | |
| S.N | **Questions** | | | | **Responses** | | | | | **Skip** |
| **301** | Surgical procedure performed Currently? | | | | 1. No surgical procedure 2. Caesarian section 3. Surgical debridement 4. External fixation 5. Appendectomy 6. Laparotomy 7. Amputation 8. OthersSpecify _________________ | | | | | Skip to 313 if ‘1’ |
| **302** | Type of surgery: | | | | 1. Clean surgery 2. Clean contaminated surgery 3. Contaminated surgery 4. Dirty surgery | | | | |  |
| **303** | Nature of surgery | | | | 1. Emergency surgery 2. Elective surgery | | | | |  |
| **304** | Preoperative hair removal | | | | 1. Previous night before surgery 2. Morning of surgery 3. During surgery 4. Not removed at all | | | | |  |
| **305** | Timing of surgical antimicrobial prophylaxis | | | | 1. Before the operation 2. During operation 3. After operation 4. Not initiated at all | | | | |  |
| **306** | Type of surgical antibiotic prophylaxis given | | | | 1. Ceftriaxone 2. Gentamycin 3. Metronidazole 4. Ampicillin 5. Others | | | | |  |
| **307** | Duration of operation in minutes | | | | 1. 0-60 min 2. 61-120 min 3. >120 min | | | | |  |
| **308** | Preoperative diagnosis | | | | 1. ________________________ 2. ________________________ | | | | |  |
| **309** | Antiseptics for Skin preparation (obtained from hospital OR) | | | | 1. Isopropyl alcohol + chlorohexidine, 2. Isopropyl alcohol + detergent, 3. Isopropyl alcohol + Iodine ) | | | | |  |
| **310** | Postoperative diagnosis | | | | 1. ________________________ 2. ________________________ 3. ________________________ | | | | |  |
| **311** | Surgeon note: | | | | __________________________________________________________________________________________________________________________________________ | | | | |  |
| **312** | Level of education of surgeon | | | | 1. General practitioner 2. Resident 3. Emergency surgeon 4. General surgeon 5. Sub-specialist surgeon | | | | |  |
|  |  | | | Vital signs | | Result | |  | | |
| **313** | Vital sign | | | 1. Blood Pressure | | ________MmHg | |  | | |
|  |  |  |  | 1. Temperature | | ________^o^c | |  | | |
|  |  |  |  | 1. Pulse Rate | | ________/min | |  | | |
|  |  |  |  | 1. Respiratory Rate | | ________/min | |  | | |
|  |  |  |  | 1. Body Mass Index | | ________kg/m^2^ | |  | | |
|  | Observations | | |  | | 1. Yes | 1. No |  | | |
| **314** | Did the patient on any one of the following devices | | | 1. Central catheter | |  |  |  | | |
|  |  |  |  | 1. Peripheral catheter | |  |  |  | | |
|  |  |  |  | 1. Uethral catheter | |  |  |  | | |
|  |  |  |  | 1. Chest tube | |  |  |  | | |
|  |  |  |  | 1. Nasog tube | |  |  |  | | |
|  |  |  |  | 1. Iv line | |  |  |  | | |
|  |  |  |  | 1. Intubation | |  |  |  | | |
|  |  |  |  | 1. Fracture fixatives | |  |  |  | | |
| **315** | For how long did the device stay in side the patient | | | 1. One day 2. Less than 3 days 3. Lessthan 7 days 4. More than 7 dyas | | | |  | | |
| **316** | For what purpose did the device insertd | | | 1. Medication Pupose 2. therapeutic service 3. Surgical preparation | | | |  | | |
| **317** | Did the patient currently taken antibiotics | | | 1. Yes 2. No | | | |  | | |
| **318** | For how long have been on such antibiotics | | | 1. <=7 days 2. 8-15 days 3. > 15 days | | | |  | | |
| **319** | For what purpose had antibiotics taken | | | 1. Prophylaxis 2. Treatment | | | |  | | |
| **320** | Route of administration | | | 1. Oral 2. Parenteral 3. Rectal 4. Inhalation | | | |  | | |

## Appendix 1.5. Checklist for assessment of Environmental Risk factors & IPPS

| Checklist for assessment of Environmental Risk factors and IPPS among staffs | | | | |
| --- | --- | --- | --- | --- |
| S.no | **Queations** | **Response** | | **Skip** |
| 501 | Hospital size (Number of Beds) | | ________________ |  |
| 502 | Number of beds per room | | ________________ |  |
| 503 | Number of beds in ICU | | ________________ |  |
| 504 | Number of admission/discharge/day | | ________________ |  |
| 505 | Alcoholic hand rub consumption/day | | ________________ |  |
| 506 | Number of Single patient room in the ward | | ________________ |  |
| 507 | Is there functional Infection prevention committee in the hospital | | 1. Yes 2. No |  |
| 508 | If ‘yes’ who are members of the committee | | _______________________  _______________________ |  |
| 508 | Number of health care worker trained for Infection prevention and personal safety from each discipline | | _______________________  _______________________ |  |
| 509 | Is there sufficient ventilation in the ward | | 1. Yes 2. No |  |
| 510 | How often was the bed made | | 1. Twice a day 2. Once a day 3. When visible soiling was seen 4. When new patient admitted |  |
| 511 | Is there enough alcohol hand rub for staffs | | 1. Yes 2. No |  |
| 512 | Is there functional sterilizing instrument in in each ward including Operation Room, Emergency, laboratory etc. | | 1. Yes 2. No |  |
| 513 | Did the staff take Regular in-service training for appropriate healthcare personnel on techniques and procedures for device (urinary catheter, Intubation, central, peripheral line catheter) insertion, maintenance, and removal | | 1. Always 2. Some times 3. Never |  |
| 514 | Did supplies necessary for aseptic device insertion Readily available | | 1. Always 2. Some times 3. Never |  |
| 515 | Urinary catheters removed postoperatively within 24-48 hours unless there are appropriate indications for continued use | | 1. Always 2. Some times 3. Never |  |
| 516 | Urinary catheters inserted using aseptic  technique and sterile equipment | | 1. Always 2. Some times 3. Never |  |
| 517 | Catheters changed at routine, fixed intervals | | 1. Always 2. Some times 3. Never |  |
| 518 | Urinary drainage bags kept below level of  bladder | | 1. Always 2. Some times 3. Never |  |
| 519 | Who inserts urinary catheters at your facility (please check all that apply)? | | 1. Registered Nurses 2. Nurse aides 3. Physicians 4. Residents 5. Intern students |  |
